# Price Analysis of Systemic Therapies and Transarterial Radioembolization for Treatment of Unresectable Hepatocellular Carcinoma

**DOI:** 10.3390/jmahp13020025

**Published:** 2025-05-27

**Authors:** Abimbola O. Williams, Nicholas Anderson, Young-Gwan Gwon, Wendy Wifler

**Affiliations:** Boston Scientific, Marlborough, MA 01752, USA; nicholas.anderson2@bsci.com (N.A.); younggwan.gwon@bsci.com (Y.-G.G.); wendy.wifler@bsci.com (W.W.)

**Keywords:** liver cancer, hepatocellular carcinoma, transarterial radioembolization, systemic therapy, treatment cost variability, price analysis

## Abstract

Systemic therapy (ST) and transarterial radioembolization (TARE) are widely used treatments for advanced-stage hepatocellular carcinoma (HCC). This study quantified the significant variability in treatment costs for unresectable HCC from payer and provider perspectives. An Excel-based price analysis model was developed to estimate the prices of ST and TARE over a 21-month time horizon using 2015–2021 data. Median prices were calculated from Medicare Average Sales Price (ASP), provider Wholesale Acquisition Cost (WAC), and Average Wholesale Price (AWP). Sensitivity analyses evaluated price fluctuations associated with a ±10% variation in treatment duration. ST prices demonstrated marked variability across perspectives, with the median ASP at $175,625, WAC at $198,719, and AWP at $262,892. However, TARE prices were stable, ranging from $21,594 to $24,052. Sensitivity analyses revealed that treatment duration variation resulted in price changes of $35,000–$50,000 for ST, compared with ~$5000 for TARE. The variability in ST pricing was driven by treatment duration and drug-specific pricing mechanisms, particularly immunotherapy-based regimens, which accounted for the higher cost range. Conversely, TARE’s consistent pricing is attributed to standardized procedural costs. Substantial variability exists in ST prices compared with the consistent costs of TARE, underscoring the economic advantage of TARE in appropriate clinical contexts.

## 1. Introduction

Liver cancer is one of the most prevalent malignancies globally and remains a leading cause of cancer-related mortality worldwide [1]. In the United States, approximately 42,230 new liver cancer cases and 30,230 liver cancer deaths were reported in 2021 [2]. Hepatocellular carcinoma (HCC), the dominant histologic subtype, accounts for 75% to 90% of all liver cancers [1,3,4]. Despite advances in screening and treatment, the incidence of HCC is projected to rise significantly, reaching 21.2 per 100,000 person-years by 2030—a 120% increase since 2013 [5]. Patients with liver cancer face a dismal 20% five-year survival rate [2], underscoring the urgent need for effective and accessible treatment strategies.

The economic burden of HCC is equally substantial. Annual direct costs range from $29,354 to $58,529 per patient [6], with additional indirect costs of $3553 per patient annually due to lost productivity [6]. The total annual cost of HCC in the United States (US) is estimated at $455 million, with indirect costs accounting for 11% of this total [6]. A critical driver of overall treatment costs is the price of the therapeutic approach. These costs vary depending on the perspective—payer versus provider—and can significantly influence resource allocation and decision-making.

For advanced-stage HCC, systemic therapy (ST) is a primary treatment modality recommended by the National Comprehensive Cancer Network (NCCN) guideline [7]. First-line systemic options include combination regimens such as atezolizumab plus bevacizumab, as well as sorafenib or Lenvatinib monotherapy [8,9]. Other approved agents include nivolumab, cabozantinib, regorafenib, ramucirumab, and FOLFOX (leucovorin, fluorouracil (5-FU), and oxaliplatin (Eloxatin)), ipilimumab, and pembrolizumab [7]. Randomized controlled trials (RCTs) have demonstrated that these therapies improve overall and progression-free survival. Transarterial radioembolization (TARE), a locoregional therapy delivered over a short duration, was selected as the comparator to ST due to its increasing adoption in clinical practice. In the US, the proportion of HCC cases treated with TARE increased from 13% in 2010 to 37% in 2017 [9,10], reflecting increased provider familiarity and broader availability. TARE is typically delivered over a short duration and may serve as an alternative or adjunct to ST in select patients [11,12].

Despite the clinical relevance of both ST and TARE, few studies have comprehensively examined their costs and how these vary by treatment modality and survival outcomes [6]. Understanding the inherent variability in treatment pricing across payer and provider perspectives is critical for making cost-conscious therapeutic decisions and optimizing resource allocation.

A primary rationale for this study was to quantify and compare the raw prices of STs and TARE, without including additional downstream costs such as imaging, follow-up care, or hospitalization. Rather than aiming for a comprehensive cost-effectiveness evaluation, this study was designed to isolate and assess variability in treatment pricing alone. This approach highlights the extent to which drug and device pricing alone can influence payer decisions, budget planning, and the overall economic burden of care. Therefore, the objective of this study is to estimate and compare the prices of ST and TARE for the treatment of advanced-stage or unresectable HCC from Medicare and provider perspectives.

## 2. Materials and Methods

### 2.1. Study Design

An Excel-based price analysis model using Microsoft^®^ Excel^®^ (Microsoft, Redmond, WA, USA) was developed to estimate the US prices of ST and TARE for the treatment of HCC from Medicare payer and provider perspectives over a 21-month time horizon.

Eleven STs and two TARE modalities were analyzed, with the median OS for STs ranging from 5.1 to 19.2 months. To ensure comparability and capture the full cost implications across the longest observed treatment durations, a 21-month time horizon was selected. This period corresponds to the longest median overall OS reported for ST in pivotal clinical trials (Table 1). The chosen duration ensures that cost estimates adequately reflect treatment persistence in longer-surviving patients on continuous ST regimens, while remaining appropriate for TARE, which is typically delivered as a fixed-course, procedure-based therapy. Although alternative durations such as 12 or 36 months could theoretically be modeled, shorter time horizons risk underestimating the cumulative ST costs, and longer horizons could introduce projection uncertainty. Thus, 21 months provide a clinically grounded and economically appropriate benchmark for estimating costs from both payer and provider perspectives.

**Figure 1:**
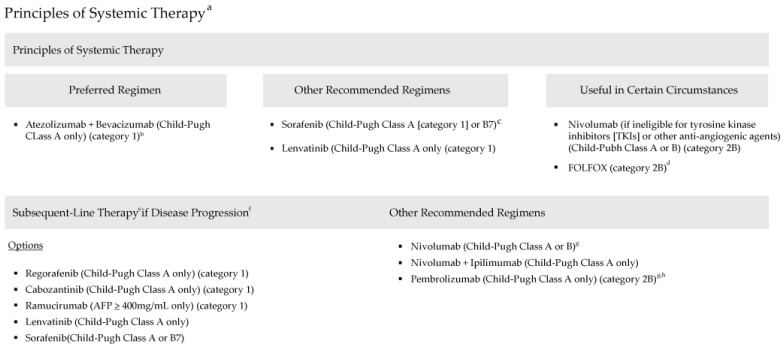
National Comprehensive Cancer Network Clinical Practice Guidelines in Oncology: Hepatobiliary Cancers (Version 2.2021). (a) See www.NCCN.org for the full recommendations (b) Patients on atezolizumab + bevacizumab should have adequate endoscopic evaluation and management for esophageal varices within approximately 6 months prior to treatment or according to institutional practice and based on the assessment of bleeding risk. (c) Caution: There are limited safety data available for Child-Pugh Class B or C patients and dosing is uncertain. Use with extreme caution in patients with elevated bilirubin levels [31]. The impact of sorafenib on patients potentially eligible for transplant is unknown. (d) There are limited data supporting the use of FOLFOX, and use of chemotherapy in the context of a clinical trial is preferred [32]. (e) Lenvatinib and entrectinib are treatment options for patients with hepatocellular carcinoma that is NTRK gene fusion positive [33,34]. (f) There are no data to define optimal treatment for those who progress after first-line systemic therapy, other than sorafenib or nivolumab. (g) For patients who have not been previously treated with a checkpoint inhibitor because there is a lack of data for subsequent use of immunotherapy in patients who have previously been treated with a checkpoint inhibitor. (h) Consider if MSI-H HCC. Adapted with permission from the NCCN Clinical Practice Guidelines in Oncology (NCCN Guidelines ©) for Hepatobiliary Cancers V.2.2021. © 2021 National Comprehensive Cancer Network, Inc. All rights reserved. The NCCN Guidelines © and illustrations herein may not be reproduced in any form for any purpose without the express written permission of the NCCN. To view the most recent and complete version of the NCCN Guidelines, go online to NCCN.org. The NCCN Guidelines are a work in progress that may be refined as often as new significant data becomes available. NCCN makes no warranties of any kind whatsoever regarding their content, use or application and disclaims any responsibility for their application or use in any way.

The model utilized data from 2015 to 2021 and followed the National Comprehensive Cancer Network^®^ Clinical Practice Guidelines in Oncology (NCCN Guidelines^®^) for Hepatobiliary Cancers, Version 2.2021 (Figure 1), which outline evidence-based recommendations for managing hepatobiliary cancers. These guidelines provide comprehensive treatment strategies for liver, gallbladder, and bile duct cancers, including locoregional therapies and systemic therapies for unresectable or advanced-stage HCC.

The price calculations in the model used several input variables (Table 1). The first was the defined daily dosage (DDD), which represents the average maintenance dose per day for a drug used for its main indication in adults [13]. The total price per DDD was calculated for each treatment therapy (ST vs. TARE) over the time horizon. Data for ST were sourced from FDA-approved drug labels listed in the FDA Orange Book and FDA Purple Book, as well as supporting RCTs [14,15].

Median overall survival (OS), defined as the length of time from either the date of diagnosis or the start of treatment for a disease, was used as a proxy for ST treatment duration. Median OS data were derived from RCTs of HCC treatments [16,17,18,19,20,21,22,23]. For TARE, the average number of devices used per procedure was based on published literature [24]. This analysis did not account for scenarios where patients might receive both therapies (e.g., incomplete response to TARE followed by ST). Ancillary costs such as hospitalization, imaging, or follow-up care were excluded to focus solely on the raw prices of the treatments.

Treatment prices were calculated using three distinct pricing metrics: average sales price (ASP), wholesale acquisition cost (WAC), and average wholesale price (AWP). Each pricing metric serves a different real-world function. ASP is primarily used for Medicare reimbursement calculations; WAC is often referenced in wholesaler purchasing contracts; and AWP is commonly cited in private payer reimbursement models. By including all three, this study provides a comprehensive assessment relevant to multiple healthcare stakeholders. The ASP was obtained from the US Centers for Medicare and Medicaid Services 2021 ASP Drug Pricing Files [25], representing prices as of April 2021. Meanwhile, the WAC and AWP were obtained from the IBM^®^ Micromedex^®^ RED BOOK^®^ Online [26], reflecting prices as of May 2021. For TARE, price estimates were drawn from published literature [27], Hospital Outpatient Prospective Payment System (OPPS), Ambulatory Surgical Center coding guides (ASC) [28,29], and from the Decision Resources Group (now Clarivate^TM^) ASP (DRG ASP) [30].

This analysis focused solely on the raw treatment prices and intentionally excluded costs related to administration, imaging, hospitalization, and follow-up. This design choice was made to isolate and analyze the inherent price variability between therapies, separate from facility-level and patient management costs. While a full cost-effectiveness model would incorporate these additional elements, our objective was to examine and compare the direct treatment price inputs that serve as key drivers in broader health economic evaluations.

**Table 1:** Input parameters.

	Length of Treatment (in Months), Based on Median Overall Survival				Cost Parameters (in USD)		
	Minimum Scenario *	Base Case	Maximum Scenario *	References	ASP [25]	WAC [26]	AWP [26]
ST							
Atezolizumab	17.3	19.2	21.1	Chiang et al., 2021 [23]	$78.15	$9469.85	$11,363.82
Bevacizumab	17.3	19.2	21.1	Chiang et al., 2021 [23]	$72.51	$796.94	$956.33
Sorafenib	11.5	12.8	14.1	Kudo et al., 2018 [22]	$174.00	$20,760.00	$24,912.00
Lenvatinib	12.2	13.6	15.0	Kudo et al., 2018 [22]	$17,099.32	$19,687.00	$23,624.40
Nivolumab	14.8	16.4	18.0	Yau et al., 2020 [21]	$28.49	$6679.16	$8014.99
Cabozantinib	9.2	10.2	11.2	Soto-Perez-de-Celis et al., 2019 [20]	$17,218.10	$21,662.80	$25,995.36
Regorafenib	9.5	10.6	11.7	Bruix et al., 2017 [19]	$17,234.43	$18,747.12	$22,496.54
Ramucirumab	7.7	8.5	9.4	Zhu et al., 2015 [18]	$5699.01	$6158.25	$7389.90
FOLFOX	5.8	6.4	7.0	Goyal, 2019 [17]	$5.33	$296.32	$1058.02
Ipilimumab	4.6	5.1	5.6	Yau et al., 2020 [21]	$7359.18	$7613.93	$9136.72
Pembrolizumab	12.5	13.9	15.3	Finn et al., 2020 [16]	$51.62	$5033.68	$6040.42
	Mean number of procedures used			References	Ljuboja et al., 2021 [27]	Medicare (OPPS/ASC) [28,29]	DRG ASP [30]
TARE							
TheraSphere	1.2	1.28	1.4	Walton et al., 2020 [24]	$18,790.57	$17,397.64	$17,088.00
SIR-Spheres	1.2	1.28	1.4	Walton et al., 2020 [24]	$18,790.57	$17,397.64	$16,653.00

ASC = Ambulatory Surgical Center; ASP = average sales price; AWP = average wholesale price; DRG = Decision Resources Group; FOLFOX = 5-FU (fluorouracil, leucovorin, and oxaliplatin); ST = systemic therapy; TARE = transarterial radioembolization; WAC = wholesale acquisition cost. * Minimum and maximum scenarios are 10% lower and 10% higher, respectively, than the base case.

### 2.2. Study Outcomes

The primary outcome was the estimated price of ST and TARE based on a typical guidelines-informed treatment regimen. Prices were calculated using multiple approaches. Firstly, the calculated maximum allowed billable units were estimated using the Medicare HCPCS code and FDA-recommended dosages. Secondly, for therapies such as bevacizumab, ramucirumab, ipilimumab, and FOLFOX, prices were adjusted based on average weight (in kg) and body surface area (BSA), sourced from the Centers for Disease Control and Prevention (CDC). This was then applied to the maximum dosage per administration [35]. Finally, for the price per DDD, weekly and monthly prices were calculated for each therapy across three pricing scenarios: Medicare, RED BOOK WAC, and RED BOOK AWP. Median and interquartile ranges (IQR) were reported for each treatment.

### 2.3. Sensitivity Analysis

A sensitivity analysis was conducted to evaluate how uncertainties in treatment duration could affect overall price estimates. To simulate real-world fluctuations in therapy duration, the median OS for ST and the average number of devices used for TARE were varied by ±10%. These adjustments generated minimum and maximum cost scenarios, providing insight into the robustness and sensitivity of the pricing estimates. This approach allowed for the evaluation of how changes in treatment persistence could affect total costs from both payer and provider perspectives, offering a practical framework for interpreting cost variability in clinical settings.

## 3. Results

The base case analysis demonstrated significant variability in ST prices per DDD across different pricing perspectives (Table 2). For individual systemic therapies, bevacizumab had the highest estimated cost across all scenarios, with a base case price of $343,156 (WAC) and $411,788 (AWP). Meanwhile, sorafenib and lenvatinib exhibited mid-range costs, with sorafenib’s base-case price ranging from $177,828 (Medicare ASP) to $318,251 (AWP), and lenvatinib’s ranging from $232,551 (Medicare ASP) to $321,292 (AWP).

The estimated prices of ST and TARE varied across the three pricing scenarios—Medicare ASP, WAC, and AWP. The median price per DDD was $175,625 (interquartile range [IQR]: $485–$232,551) for Medicare ASP, $198,719 (IQR: $52,345–$343,156) for WAC, and $262,892 (IQR: $62,814–$411,788) for AWP across all pricing scenarios (Table 3). In contrast, TARE had a substantially lower cost across all scenarios, with a median price ranging from (excluding consumables) $24,052, the Medicare ASP was $22,269, and the provider ASP was $21,594 (Table 3).

Given that the price of ST is directly influenced by treatment duration, variations in median OS resulted in fluctuations in total cost. Across the range of estimated OS values (5.1–19.2 months for ST and 1.28–1.4 procedures for TARE), the cost of ST increased by approximately 10% in the maximum scenario, reaching a peak of $289,181 (AWP). In contrast, TARE costs remained more stable, with the maximum scenario price reaching $26,457 (Table 3).

**Figure 2:**
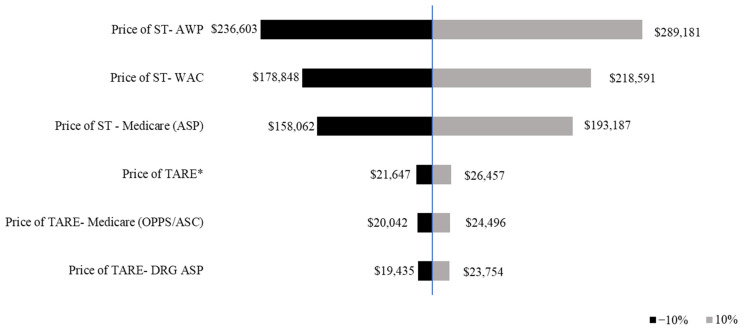
Tornado diagram of one-way sensitivity analysis of the median price of systemic therapy and transarterial radioembolization. The prices of ST and TARE are shown in 2021 USD. The tornado diagram shows the one-way sensitivity analysis results, with the black bars indicating the price of ST/TARE when the median overall survival is varied by −10%, thus, representing assumptions below the base case analysis. The gray bars indicate the mean price of ST/TARE when the median overall survival is varied by +10%. * Shows Price of TARE using information from Ljuboja et al., 2021 [27].

The sensitivity analysis assessed the impact of a ±10% variation in treatment duration on median prices. The price of ST exhibited a wider range of variation compared with TARE, indicating a greater sensitivity to changes in treatment duration. A 10% decrease in median OS resulted in lower costs across all pricing models, with ST declining from $218,591 (WAC) to $193,187 (Medicare ASP), and TARE from $24,496 to $23,754. A 10% increase in median OS resulted in higher total treatment costs, with ST peaking at $289,181 (AWP), while TARE remained relatively stable at $26,457 (Figure 2).

## 4. Discussion

This study highlights substantial variability in the estimated prices of ST and TARE for HCC based on different pricing perspectives. Systemic therapies demonstrated substantially higher and more variable costs compared with the pricing of TARE. Specifically, the mean Medicare ASP for ST ($147,523) was less than 60% of the mean provider AWP ($249,107), underscoring the influence of perspective on pricing. Within each perspective, ST costs varied widely due to differences in treatment duration (median OS) and individual drug pricing mechanisms, as reflected by the large IQRs.

The findings align with previous research emphasizing the dual importance of drug effectiveness and cost in determining the overall cost-effectiveness of ST. For example, cost-effectiveness analyses of ST have shown that both drug price and survival outcomes are critical to selecting first-line therapies for HCC [23,36]. The relatively predictable pricing of TARE, requiring fewer visits and procedures than ST, offers an economic advantage for resource-constrained settings or healthcare systems prioritizing cost containment.

Previous studies have examined the costs of ST and TARE. Marqueen et al. demonstrated a higher monthly drug price for sorafenib ($78,859) than TARE ($58,397) using Medicare reimbursement data [37]. The authors included costs associated with monitoring and follow-up with outpatient computerized tomography scans every three months, which is not the focus of the current analysis. Rather, this analysis focused on the raw prices of the treatments. Similarly, Manas et al. used a Markov model to evaluate TARE costs (£48,583, equivalent to $66,559 using a 2021 currency conversion rate of 1.37) in a UK population [38]. However, it may not be appropriate to extrapolate the UK study results to the US population due to differences in population characteristics, treatment patterns, and methodologies.

Given the high cost of cancer therapies for treating HCC, the potential implications of these results are numerous. The financial burden of ST can extend beyond direct drug costs to include frequent office visits, laboratory tests, imaging, and associated services. For patients requiring sequential or combined therapies, costs may escalate further depending on disease progression and treatment tolerance. Expanding access to TARE therapies, such as TheraSphere™, could reduce overall treatment costs due to fewer required procedures and stable pricing. Moreover, studies have documented the clinical benefits of TARE across early, intermediate, and advanced HCC stages, suggesting potential for both economic and clinical gains [39,40,41,42].

This study has several strengths. It provides a comparative analysis of ST and TARE pricing perspectives, incorporating real-world treatment scenarios aligned with clinical guidelines. The findings directly apply to healthcare decision-making, enabling clinicians and policymakers to balance costs with clinical efficacy. However, several limitations warrant consideration.

The analysis focused solely on raw prices, excluding administration, hospitalization, and mapping costs for TARE, which may underestimate total direct costs. The study did not account for patient-level factors such as copayments, rebates, or insurance-specific coverage variations that could influence individual costs. Additionally, there is likely variability in the price of STs due to differences in prescription filling and health plan coverage. Although the IBM RED BOOK was used to estimate drug prices for WAC and AWP, these prices may not accurately reflect the cost for individual patients.

Additionally, the therapeutic landscape for HCC continues to evolve, with emerging treatments such as tremelimumab plus durvalumab used instead of sorafenib as a first-line treatment from the HIMALAYA RCT, which demonstrates significant clinical benefits, but it has not yet been widely integrated into guidelines [43]. Also, the NCCN guidelines do not reflect all recent developments. Certain therapies listed in the NCCN guidelines may not reflect real-world prescribing trends. For instance, FOLFOX is infrequently used in clinical practice, and nivolumab may be used off-label or have been withdrawn by its manufacturer. Moreover, the results presented for TARE reflect a scenario in which this therapy was 100% effective for patients. Estimates of the proportion of patients who experience this vary, although multiple studies report that the percentage of those who experience a complete response ranges between 31% and 59% [44,45,46], depending on patient selection and tumor characteristics. Patients who experience incomplete responses to TARE may require subsequent ST, which could significantly alter cost projections. Therefore, the results may not be applicable to all TARE patients, but only to those who experience 100% effectiveness of TARE.

This study did not account for unknown drug rebates or patient assistance programs negotiated directly with manufacturers by benefit management entities. Additionally, this research did not account for certain patient-level differences in insurance coverage, rebates, or copayments for systemic medical therapy that may be subject to manufacturer-patient assistance programs or medical benefits that cover interventional outpatient treatments. Also, this study includes only direct treatment-related costs, and does not account for indirect costs such as lost productivity, caregiver burden, or transportation—factors that can materially affect patient-level financial burden and societal costs. While the 21-month timeframe was chosen to align with the longest median overall survival (19.2 months), cost-effectiveness analyses often select annual horizons, such as 12 or 36 months, which we did not consider. Finally, this analysis was conducted using version 2.2021 of the NCCN guidelines, which was the most recent version at the time of analysis. An updated NCCN clinical practice guideline was published in September 2021 (version 5.2021). However, there were no significant changes to the recommended therapy for HCC, so the price differences between versions would be minimal. Nevertheless, newer therapies that have since gained approval or adoption were not included in this model, and future studies should incorporate them as survival and pricing data become available.

Despite these limitations, this study provides valuable real-world evidence on the raw prices of HCC therapies and highlights the wide variability in pricing by treatment option and perspective. Clinicians should carefully consider the potential clinical benefits and risks when selecting an appropriate therapy for their patients. Understanding the potential economic implications of different therapies can help providers and payers make informed decisions on cancer therapies. Future research should integrate cost-effectiveness analyses to evaluate ST and TARE in combination or as sequential treatments, incorporating both direct and indirect costs. Real-world studies exploring the impact of drug rebates, patient assistance programs, and insurance coverage variations would provide a more comprehensive view of patient-level financial burdens.

## 5. Conclusions

This study underscores the significant variability in ST pricing compared with TARE for advanced-stage or unresectable HCC. These findings provide actionable insights for clinicians, policymakers, and healthcare stakeholders, highlighting the importance of aligning treatment selection with both clinical effectiveness and economic considerations. These findings can inform resource allocation, improve patient access to cost-effective therapies, and support efforts to optimize HCC management strategies.

## Figures and Tables

**Table 2:** Price analysis of systemic therapy and selective internal radiation therapy for the treatment of hepatocellular carcinoma.

**Treatment Option**	Minimum Scenario *			**Base Case**			Maximum Scenario *		
	Medicare ASP [25]	WAC [26]	AWP [26]	Medicare ASP [25]	WAC [26]	AWP [26]	Medicare ASP [25]	WAC [26]	AWP [26]
ST									
Atezolizumab	$162,058	$163,639	$196,367	$180,065	$181,821	$218,185	$198,071	$200,003	$240,004
Bevacizumab	$96,484	$308,840	$370,609	$107,205	$343,156	$411,788	$117,925	$377,472	$452,967
Sorafenib	$160,045	$238,688	$286,426	$177,828	$265,209	$318,251	$195,611	$291,730	$350,076
Lenvatinib	$209,296	$240,969	$289,163	$232,551	$267,743	$321,292	$255,806	$294,518	$353,421
Nivolumab	$201,824	$197,169	$236,603	$224,250	$219,076	$262,892	$246,675	$240,984	$289,181
Cabozantinib	$158,062	$198,865	$238,637	$175,625	$220,961	$265,153	$193,187	$243,057	$291,668
Regorafenib	$164,416	$178,848	$214,617	$182,685	$198,719	$238,463	$200,953	$218,591	$262,310
Ramucirumab	$43,597	$47,111	$56,533	$48,442	$52,345	$62,814	$53,286	$57,580	$69,096
FOLFOX	$436	$62,524	$258,324	$485	$69,471	$287,027	$533	$76,419	$315,730
Ipilimumab	$135,115	$139,791	$167,750	$150,127	$155,324	$186,389	$165,140	$170,856	$205,027
Pembrolizumab	$129,143	$125,943	$151,131	$143,492	$139,936	$167,924	$157,842	$153,930	$184,716
TARE	Ljuboja et al., 2021 [27]	Medicare (OPPS/ASC) [28,29]	DRG ASP [30]	Ljuboja et al., 2021 [27]	Medicare (OPPS/ASC) [28,29]	DRG ASP [30]	Ljuboja et al., 2021 [27]	Medicare (OPPS/ASC) [28,29]	DRG ASP [30]
TheraSphere	$21,647	$20,042	$22,269	$24,052	$22,269	$21,873	$26,457	$24,496	$24,060
SIR-Spheres	$21,647	$20,042	$22,269	$24,052	$22,269	$21,316	$26,457	$24,496	$23,447

ASC = Ambulatory Surgical Center; ASP = average selling price; AWP = average wholesale price; DRG = Decision Resources Group; FOLFOX = 5-FU (fluorouracil, leucovorin, and oxaliplatin); TARE = transarterial radioembolization; WAC = wholesale acquisition cost. * Minimum and maximum scenarios are 10% lower and 10% higher, respectively, than the base case.

**Table 3:** Descriptive summary of the price of systemic therapy and selective internal radiation therapy for the treatment of hepatocellular carcinoma.

		Minimum Scenario *			Base Case			Maximum Scenario *		
		Medicare ASP [25]	WAC [26]	AWP [26]	Medicare ASP [25]	WAC [26]	AWP [26]	Medicare ASP [25]	WAC [26]	AWP [26]
Systemic Therapy	Mean Cost	$132,771	$172,944	$224,196	$147,523	$192,160	$249,107	$162,275	$211,376	$274,018
	Median Cost	$158,062	$178,848	$236,603	$175,625	$198,719	$262,892	$193,187	$218,591	$289,181
		Ljuboja et al., 2021 [27]	Medicare (OPPS/ASC) [28,29]	DRG ASP [30]	Ljuboja et al., 2021 [27]	Medicare (OPPS/ASC) [28,29]	DRG ASP [30]	Ljuboja et al., 2021 [27]	Medicare (OPPS/ASC) [28,29]	DRG ASP [30]
TARE	Mean Cost	$21,647	$20,042	$19,435	$24,052	$22,269	$21,594	$26,457	$24,496	$23,754
	Median Cost	$21,647	$20,042	$19,435	$24,052	$22,269	$21,594	$26,457	$24,496	$23,754
		Medicare ASP [25]	WAC [26]	AWP [26]	Medicare ASP [25]	WAC [26]	AWP [26]	Medicare ASP [25]	WAC [26]	AWP [26]
IQR	FOLFOX	$436			$485			$533		
	Lenvatinib	$209,296			$232,551			$255,806		
	Ramucirumab		$47,111	$56,533		$52,345	$62,814		$57,580	$69,096
	Bevacizumab		$308,840	$370,609		$343,156	$411,788		$377,472	$452,967

ASC = Ambulatory Surgical Center; ASP = average selling price; AWP = average wholesale price; DRG = Decision Resources Group; FOLFOX = 5-FU (fluorouracil, leucovorin, and oxaliplatin); TARE = transarterial radioembolization; WAC = wholesale acquisition cost. * Minimum and maximum scenarios are 10% lower and 10% higher, respectively, than the base case.

## Data Availability

This study used data from IBM, CMS, and published studies. Due to data use agreements signed with IBM Watson, the data cannot be provided externally. Other researchers can purchase the same dataset to conduct similar analyses. Data from CMS is freely accessible online.

## Abbreviations

The following abbreviations are used in this manuscript:

5-FU	5-Fluorouracil
ASP	Average Sales Price
AWP	Average Wholesale Price
BCLC	Barcelona Clinic Liver Cancer
BMS	Bare-Metal Stent
CDC	Centers for Disease Control and Prevention
DCB	Drug-Coated Balloon
DDD	Defined Daily Dosage
DES	Drug-Eluting Stent
DRG ASP	Decision Resources Group Average Sales Price
FDA	Food and Drug Administration
FOLFOX	Leucovorin, Fluorouracil (5-FU), and Oxaliplatin
HCC	Hepatocellular Carcinoma
HCPCS	Healthcare Common Procedure Coding System
IQR	Interquartile Range
MeSH	Medical Subject Headings
NCCN	National Comprehensive Cancer Network
OPPS	Hospital Outpatient Prospective Payment System
OS	Overall Survival
PTA	Percutaneous Transluminal Angioplasty
RCT	Randomized Controlled Trial
ST	Systemic Therapy
TARE	Transarterial Radioembolization
US	United States
WAC	Wholesale Acquisition Cost
